# Identification of a Novel Stop Loss Mutation in *P2RX2* Gene in an Iranian Family with Autosomal Nonsyndromic Hearing Loss

**DOI:** 10.52547/ibj.25.5.368

**Published:** 2021-07-13

**Authors:** Reza Azizi Malamiri, Javad Mohammadi Asl, Farideh Ghanbari

**Affiliations:** 1 Department of Pediatrics, Faculty of Medicine, Ahvaz Jundishapur University of Medical Sciences, Ahvaz, Iran;; 2 NoorGene Genetics Lab, Ahvaz, Iran;; 3 Department of Medical Genetics, School of Medicine, Tehran University of Medical Sciences, Tehran, Iran

**Keywords:** Autosomal dominant 41, Deafness, Mutation, P2RX2, Whole exome sequencing

## Abstract

**Background:**

Hearing loss, a congenital genetic disorder in human, is *difficult *to diagnose. WES is a powerful approach for ethiological disgnosis of such disorders.

**Methods:**

One Iranian family with two patients were attented in the study. Sequencing of known NSHL genes was carried out to recognize the genetic causes of HL.

**Results:**

Molecular analyses identified a novel stop loss mutation, c.1048T>G (p.Term350Glu), whitin the *P2RX2 *gene, causing a termination-site modification.This event would lead to continued translation into the 3' UTR of the gene, which in turn may result in a longer protein product. The mutation was segregating with the disease phenotype and predicted to be pathogenic by bioinformatic tools.

**Conclusion:**

This study is the first Iranian case report of a diagnosis of ADNSHL caused by *P2RX2 *mutation. The recognition of other causative mutations in *P2RX2* gene more supports the probable function of this gene in causing ADNSHL.

## INTRODUCTION

Hearing loss is socially and economically important cause of human morbidity and is the most common sensorineural  deficiency in humans. HL affects more than 300 million people worldwide^[^^1^^,^^2^^]^. Genetic factors account for more than 50% of all prelingual HL cases. Hereditary HL is mainly classified into two forms: syndromic HL (approximately 30%), in which HL is accompanied by other clinical manifestations, and NSHL (approximately 70%), in which there are no further abnormal features^[^^3^^]^. About 20–25% of NSHL forms are ADNSHL and 75–80% are ARNSHL, while only 1–1.5% is X-linked^[^^3^^]^. To date, 80 genes and up to 140 genetic loci have been identified to *be* associated with NSHL (http://hereditaryhearingloss.org/). Molecular diagnosis plays a key role in clinical management, prognosis evaluation and pre-implantation genetic diagnosis for NSHL families^[^^4^^]^.  

Until now, the extensive genetic heterogeneity of hearing impairment has restricted genetic diagnosis in most patients. Advances in DNA sequencing technologies such as NGS have facilitated the DNA testing and complete genetic analysis ofheterogeneous diseases. Targeted NGS provides a significant opportunity to detect variants in recognized disease genes, mainly in extremely heterogeneous diseases such as deafness^[^^5^^]^.

On the basis of these data, we aimed to recognize gene defects in an Iranian family with NSHL. This study reports a pathogenic mutation of *P2RX2* gene, which produces an extended protein-coding transcript that may explain the NSHL phenotype in the family.

## MATERIALS AND METHODS

**Clinical evaluations**

**Fig. 1:**
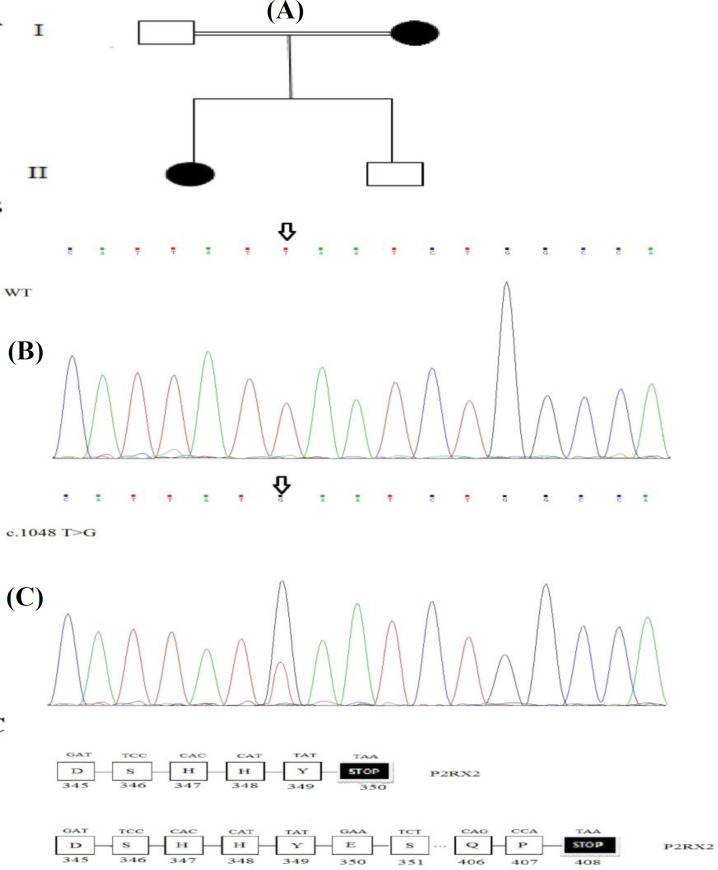
(A) Pedigree of family with ARNSHL. The c.1048T>G variant is inherited from mother to the affected proband (denoted in black). (B) Electropherograms analysis of P2RX2 in ADNSHL affected probanda c.1048T>G variant (shown with arrow).  (C) Zoomed-in view of region containing the variant, including the amino acid sequences of protein-coding isoform and the mutated sequences

**Fig. 2:**
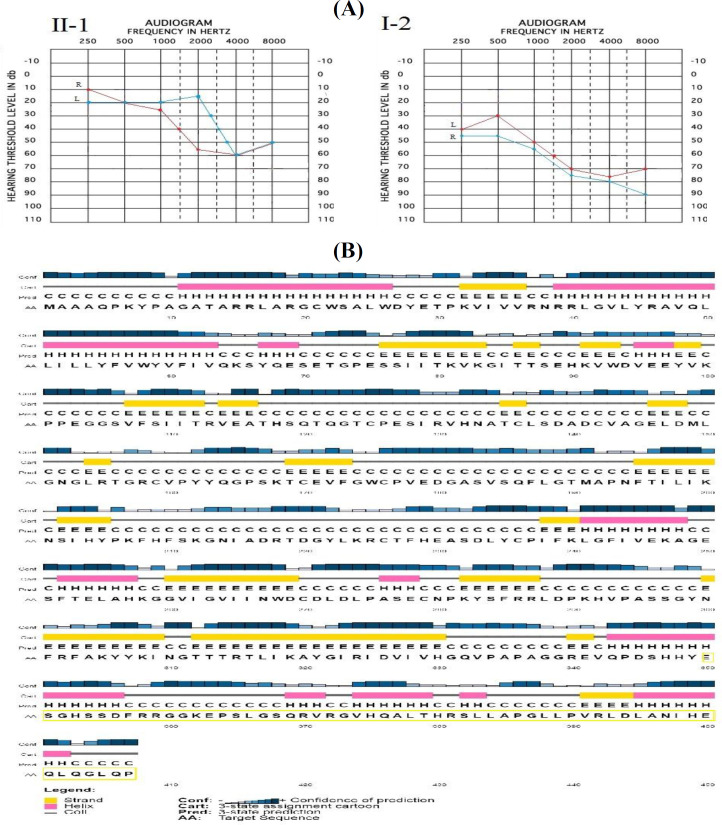
(A) Audiograms of the affected family members (L and R show left and ears, respevtively); ; (B) the predicted secondary structure of elongated mutant P2RX2 protein. The mutant P2RX2 protein is composed of new α-helices (pink) and strands (yellow), compared with the wild-type P2RX2 protein composed of 6 α-helices (data not shown). Coils are presented as straight lines, and 58 additional amino acids residues in the C-terminal highlighted in yellow generate new α-helices and strands

Two patients in a family with a bilateral, sensorineural and progressive form of ADNSHL were enrolled in the study. The index patient was an 11-year-old female, the first child of a couple with consanguineous marriage orginated from the southwest of Iran (Fig. 1A). She was diagnosed with congenital deafness and had no dysmorphic features. The age of onset in the family was in the first decade of life. There was no history of systemic disease in the proband. A full clinical explanation was obtained to exclude environmental exposures and features indicative of syndromic anomalies in the family. Also, pedigree examination, physical assessments, audiological tests (Fig. 2A), opthalmological evaluation, and electrocardiograms in the proband were carried out. 

**Molecular analysis**

Peripheral* blood samples were collected from* the patients and other member of the family, and the genomic DNA was extracted from blood samples (family members) by using standard protocols^[^^6^^]^. Libraries were prepared following standard Illumina, sample protocol. In precis, 3 µg of genomic DNA was fragmented to 200–300 bp. Terminal A residues were added following the incubation with the *Klenow Fragment exo*-(*3*'→*5*′ *exo*-) and dATP. Thereafter,

adapters were ligated to 3' and 5’ ends of the fragments. Then, the 200–300 bp product was chosen for further PCR amplification. A Human capture array (Roche NimbleGen, USA) was designed to capture all coding regions, and the intron/exon boundaries of the genes, which are involved in the pathogenesis of HL, followed by NGS approach (BGI-Shenzhen, Guangdong, China). After NGS sequencing, the sequence reads were mapped onto the reference human genomic DNA (UCSC/hg19). For the subsequent variant analysis, GATK software version 4.1 was used to assemble the consensus sequence and detect single nucleotide variants and indels in target regions. Moreover, the search for rare variants (minor allele frequency (< 1%), which were particularly found in the affected girl, was carried out by using single nucleotide polymorphism and 1000 Genomes databases. The effect of these candidate variants on protein structure and phylogenetic conservation was estimated by bioinformatic tools such as SIFT^[^^7]^, PolyPhen-2^[^^8^^]^, and Mutation Taster^[^^9^^]^, to predict the pathogenicity of variants. Potential candidate variants observed in each gene using NGS were confirmed by direct sanger sequencing with ABI3500 sequencer (Applied Biosystems, USA). PCR primer sequences and conditions are available on request. Segregation analysis was carried out for other relatives members. Also, PSIPRED 4 (http://bioinf.cs.ucl.ac.uk/psipred)^[^^10^^]^ was applied to predict the secondary structure of the wild-type *P2RX2* and elongated mutant *P2RX2* (p.*350Gluext*58).

**Ethical statement**

The above-mentioned protocols were approved by the Research Ethics Committee of Ahvaz Jundishapur University of Medical Sciences, Ahvaz, Iran (ethical code: U-91074). Written informed consents were provided by the patients and their parents.

## DISCUSSION

In present study, we recognized a novel mutation in the *P2RX2 *gene in an Iranian pedigree with NSHL. The *P2RX2 *gene is one of the most recent genes known as a cause of ADNSHL (OMIM; 608224). The *P2RX2 *mutations seem to be very rare for the reason that there have been only two described mutations: c.1057G>C (p.Gly353Arg) and c.178G>T (p.Val60Leu) from Italy and China, respectively^[^^11^^,^^12^^]^. 

The P2X2 receptor is a protein encoded by the *P2RX2 *gene and plays an important function in the cochlea as a ligand-gated ion channel receptor by ATP-mediated regulation^[^^13^^]^. This ATP-activated ion channel binding to ATP intercedes synaptic transmission between some neurons and from neurons to smooth muscle cells. P2X2 receptors are predominantly  expressed in the epithelial cells near the cochlear duct of the endolymphatic compartment in the inner ear, which consists of the organ of Corti^[^^14^^]^. The P2X2 protein is upregulated due to exposure to noise. ATP is regarded to have a neurotransmission effect at the hair cells synapse and chip in the regulation of the endocochlear potential^[^^15^^,^^16^^]^.

Each receptor is composed of three polypeptide subunits (P2X1-7), which all share the general basic structure of intracellular C- and N-terminal, a large extracellular loop and two transmembrane domains. Functional receptors can be organized from triplets of identical subunits or homomeric and can also exist as heteromers complexes^[^^17^^]^. Yan *et al.*^[^^11^^]^ have previously reported two unrelated Chinese pedigrees with autosomal dominant deafness-41, which carry a missense mutation (p.Val60Leu) in the *P2RX2* gene, causing a substitution between two hydrophobic amino acids and removing the P2X2 response to ATP. This missense mutation in the first Chinese pedigree was recognized by WES; the second unrelated Chinese pedigree was 1 of 65 pedigrees in whom the *P2RX2* gene was sequenced. Moreover, a missense mutation in the *P2RX2* gene (p.Gly353Arg) in an Italian pedigree with dominant deafness-41 was described by Faletra *et al.*^[^^12^^]^, confirming the finding of Yan *et al.*^[^^11^^]^ who implied that mutations in the *P2RX2* gene can cause progressive deafness.

The substitution of a stop codon with a charged amino acid such as glutamic acid could threaten the fold of the protein and interaction with the membrane. These results strongly propose that the p.Ter350Glu mutation should have a related impact on both the structure of the protein and function. Secondary structural study revealed that the wild-type *P2RX2* protein is consisted of six α-helices. Though the extended mutant *P2RX2* is consisted of six α-helices, the elongated 58 additional residues in C-terminal sequence produce new α-helix and β strand, finally causing the change of the structure of *P2RX2 *(Fig. 2B).

Also, multiple sequence alignments of human *P2RX2* protein by ConSeq web server (PolyPhen, SIFT, and Mutation Taster) proved high conservation of this amino acid among various types of species; hence, this mutation can affect the ATP-mediated regulation activity of P2RX2 and can cause developmental *abnormalities,*  leading to autosomal dominant deafness. Generally, it seems that this substitution could have a key function in the P2RX2 protein, and mutations at this site give rise to pathogenicity and deafness. In the present study, the modification in *P2RX2 gene* was found to interfere with the normal stop codon located at position 350 in exon 10, causing a termination-site change and continuation of translation into the 3' UTR, recognized (Fig. 1C) from one allele of proband. Stop-loss variants are single base-pair exchanges that happen within translational termination codons, which could result in the continued translation of the *messenger*  RNA into the 3′ UTR^[^^18^^]^. These variants mutations are reported in some cases involving different diseases^[^^19^^,^^20^^]^.

The p.Ter350Glu alters the *amino acid* sequence of P2RX2 protein and typically *causes* the open reading frame of the protein as the wild-type protein has 349 residues, while the mutant type has 407 amino acids. A stop loss mutation in the *P2RX2* gene (c.1048 T>G: p.Term350Glu; Fig. 1B) destroys the functional ochre *termination* codon (UAA) at the 3' end of *P2RX2*. The next *termination c*odon is a 174-bp downstream (in the 3′ UTR), expecting to add 58 residues to the carboxy-terminal end of P2RX2, though no functional analysis procedures were carried out to verify this. There are three hypotheses. First, the mRNA transcript comprising the stop loss allele is degraded (with 'non-stop decay' pathway), proposing haploinsufficiency of *P2RX2*^[^^21^^]^. The second mechanism makes the protein chains unable to assemble in the correct fashion. In this setting, the chain is not generally very stable. Third, while this process of non-stop decay is fairly effective at removing stop loss mRNAs, any protein products generated by the translation of residual stop loss mRNAs are degraded by the proteasome. Therefore quantitative- and translation-based researches are needed to prove the lack of both the mutant RNA and the extended protein. Even though a number of researchers implicitly suppose that the normal open reading frame will simply be developed until the next in-frame termination codon is encountered. Very few stop loss mutations in human have hitherto been recognized to allow any common conclusions to be drawn as to their possiblity phenotypic results  in either expression *levels* of *protein* or *mRNA*^[^^18^^]^.

In conclusion, using WES, we identified one novel stop loss mutation (p.Ter350Glu) in *P2RX2* in Iranian family members with ADNSHL. Our findings expand the *P2RX2* mutation spectrum, and the detection of additional disease-causing mutations in this gene could verify more the crucial role of the *P2RX2* in auditory function. Moreover, further functional studies are required to investigate the role of p.Ter350Glu mutation in the function of auditory.
